# Cytotoxic Effect of the Genus *Sinularia* Extracts on Human SCC25 and HaCaT Cells

**DOI:** 10.1155/2009/634868

**Published:** 2008-10-13

**Authors:** Guey-Horng Wang, Tzung-Han Chou, Rong-Jyh Lin, Jyh-Horng Sheu, Shih-Hao Wang, Chia-Hua Liang

**Affiliations:** ^1^Department of Cosmetic Science, Chia Nan University of Pharmacy and Science, Tainan 717, Taiwan; ^2^Department of Parasitology, Faculty of Medicine, Kaohsiung Medical University, Kaohsiung 807, Taiwan; ^3^Department of Marine Biotechnology and Resources, National Sun Yat-Sen University, Kaohsiung 804, Taiwan; ^4^Graduate Institute of Pharmaceutical Chemistry, China Medical University, Taichung 404, Taiwan

## Abstract

Soft corals of the genus *Sinularia* are being increasingly adopted to treat a wide variety of disease processes. However, the mechanism underlying its activity against human oral cancer cells is poorly understood. This study evaluates the cyototoxicity effects of the genus *Sinularia* extracts (*S. grandilobata, S. parva, S. triangula, S. scabra, S. nanolobata* and *S. gibberosa*) by SCC25 and HaCaT cells. The cell adhesion assay indicates that extracts reduce the cell attachment. Extracts exhibit a dose-dependent cytotoxic effect using MTS assay.Treatment of extracts to observe the morphological alterations in cells, membrane blebbing, nuclear condensation, and apoptotic bodies is demonstrated. Flow cytometry shows that extracts sensitized the cells in the G_0_/G_1_ and G_2_/M phases with a concomitant significantly increased sub-G_1_ fraction, suggesting cell death by apoptosis. Extracts of the genus *Sinularia* thus apparently cause apoptosis of SCC25 and HaCaT cells, and warrant further research investigating the possible antioral cancer compounds in these soft corals.

## 1. Introduction

Medicinal marine organisms are most appropriate for pharmacological research and drug development, since their constituents can be employed not only as therapeutic agents, but also as starting materials or models for synthesis of drugs of pharmacologically active compounds. Many efforts have recently been made to identify new therapeutic drugs against cancer, especially using novel biologically active compounds from natural marine organisms [1].

Coral growths are a few hundred million years old. Pressure from the natural selection has led corals to develop a delicate chemical balance for self protection. Soft corals (coelenterata, octocorallia, alcyonaceae) are a rich source of steroids and terpenoids [2, 3], and most isolated diterpenes are cembranolides [4]. Hence, such chemical toxins may exhibit various biological activities, such as antitumor, antimicrobial, and HIV-inhibitory activity. The authors have previously reported various bioactive metabolites from marine organisms, including sesquiterpenoids, diterpenoids, and steroids [5–8]. Many of these metabolites have been found to be cytotoxic, or to possess other biological activities [9–11]. However, little thorough cytotoxicity research has been performed on these soft corals. Therefore, this study investigates the cytotoxic mechanism of the organic extracts of six Taiwanese soft corals, namely *Sinularia grandilobata*, *S*. *parva*, *S*. *triangula*, *S*. *scabra*, *S*. *nanolobata,* and *S*. *gibberosa*.

Oral cancer is a significant global public health problem, causing high morbidity and mortality that have not improved in decades [12]. Squamous cell carcinomas (SCCs) are the most common type of oral cancer. Although new operative techniques and adjuvant measures including chemotherapy and radiotherapy against oral SCCs have progressed, patients with advanced oral SCCs still have a poor prognosis, with a 5-year survival rate of 65% [13]. Thus, new anticancer drugs are required to enhance current protocols for diagnosis and treatment of SCCs.

Apoptosis is an important phenomenon in exerting antitumor response to cancer therapy and is also a valuable marker for predicting tumor response following anticancer treatment. Cell death can be apoptotic, or result from morphological changes such as membrane blebbing, cell shrinkage, chromatin condensation, and nuclear fragmentation with formation of apoptotic bodies. Translocation of membrane phosphatidylserine and sub-G_1_ fraction is a form of programmed cell death that occurs naturally in cells and can be beneficial to cancer therapy [14]. Ability to manipulate the machinery of cell death is an obvious goal of medical research, and effect on regulation of apoptosis might lead to new possibilities for oral cancer treatment [15]. Hence, this study evaluated the induction of cell apoptosis of the genus *Sinularia* extracts on human SCC25 cells and premalignant keratinocytes (HaCaT).

## 2. Materials and Methods

### 2.1. Material

The six soft corals of the genus *Sinularia* including *S*. *grandilobata*, *S*. *parva*, *S*. *triangula*, *S*. *scabra*, *S*. *nanolobata* and *S*. *gibberosa* were collected by hand via scuba along the coast of Southern Taiwan, at a depth of 10–15 m and were stored in a freezer until extraction. A voucher specimen was deposited at the Department of Marine Biotechnology and Resources, National Sun Yat-Sen University, Taiwan.

### 2.2. Preparation of Extracts

The tissues of six soft corals of the genus *Sinularia* were freeze-dried and then exhaustively extracted with ethyl acetate (two times). The ethyl acetate extracts were then filtered and concentrated under vacuum to provide a brownish semisolid crude extract. Organic extracts were dissolved at a concentration of 10 mg/mL in 100% dimethyl sulfoxide (DMSO) at stock solution. Stock solution was diluted to the desired final concentrations with growth medium just before use. The final DMSO concentration did not exceed 0.1%.

### 2.3. Cell Lines and Cell Culture

Human oral squamous cell carcinoma (SCC25) cells was purchased from the American Type Culture Collection (Rockville, Md, USA). Human premalignant keratinocytic cells (HaCaT) were a kind gift from Hamm-Ming Sheu (National Cheng Kung University Medical College, Tainan, Taiwan). Cells were cultured in medium supplemented with 10% fetal bovine serum (Hazelton Product, Denver, Pa, USA) and 1% penicillin-streptomycin at 37°C in 5% CO_2_; specifically SCC25 cells in Dulbecco's Modified Eagle's Medium/F12 medium and HaCaT cells in Dulbecco's Modified Eagle's Medium medium (GIBCO, Grand Island, NY, USA).

### 2.4. Cell Adhesion Assay

Cells (1.5 × 10^5^ cells/well) were subcultured into 24-well plates and incubated. After 24 hours of incubation, the medium was changed by adding DMEM/F12 or DMEM containing 1% bovine serum albumin (BSA) and with or without serial concentrations ofextracts for 18 hours. Attached cell number was estimated by means of a DNA carmine-based colorimetric method [16]. Briefly, cells were fixed with 100% methanol, dried and stained with alcoholic/HCl carmine. Colorant was extracted with 0.01 N NaOH, and absorbance was determined at 540 nm. The cell number was estimated using a titration curve of cell density (SCC25: *y* = 5 × 10^−8^ *x* + 0.0143; *R* ^2^ = 0.9849; HaCaT: *y* = 3 × 10^−8^ *x* + 0.0019; *R* ^2^ = 0.9904), and results were given as a percentage of the cell number with respect to control cells. For the titration curve, cells were plated at densities ranging form 1 × 10^3^ to 7 × 10^5^ cells/well in 24-well plates using serial dilutions of concentrated cell suspensions. After adhesion, some wells of each density were harvested with trypsin and cells were counted in a hemacytometer; meanwhile, parallel cultures were fixed and stained as described before [16]. A relationship between the cell number and resultant absorbance after the colorant extraction, for each cell density, was accomplished and cell-density titration-curve construction, which measured cell adhesion.

### 2.5. Growth-Inhibition Assay

Cells (1.5 × 10^4^ cells/well) were seeded in each 100 *μ*L of 96-well multidishes for at least 24 hours prior to use. The cells were treated with serial concentrations of extracts for 18 hours. After replacing new medium, the effects on cell growth were determined by a colorimetric tetrazolium MTS [3-(4,5-di-methyl-thiazol-2-yl)-5-(3-carboxymethoxyphenyl)-2-(4-sulfophenyl)-2H-tetrazolium, inner salt] assay according to the manufacturer's procedure (CellTiter 96 AQ, Promega, Madison, Wis, USA). The absorbance at 490 nm was measured by a spectrophotometer (Dydatech, Alexandria, Va, USA). Values are expressed as the percentage of mean cell viability is relative to the untreated cultures. The IC_50_ and IC_80_ were calculated from the drug concentration that induced 50% and 80% of cell survival rate. All determinations were performed in triplicate and statistically analyzed by Student's *t*-test.

### 2.6. Determination of Morphological Changes of Cells

Cells (1.5 × 10^5^ cells/well) were plated in 24-well plates then treated with IC_50_ concentrations of extracts for 18 hours. After incubation, the medium was removed and cells were fixed in 4% paraformaldehyde and permeabilized in saponin (0.1% v/v in PBS-BSA). Morphological analysis was performed using phase contrast inverted light microscope (Nikon, TE2000-U, Japan) at 200× magnification. To assess specific apoptosis, Hoechst (1 *μ*g/mL) (Sigma, USA) was added to each well and further incubated at 37°C for 30 minutes in the dark. Living and apoptotic cells were visualized through blue filter of fluorescence inverted microscope (Nikon, TE2000-U, Japan) at 200× magnification.

### 2.7. Assessment of Cell-Cycle Distribution and Apoptotic Cells by Flow Cytometry

Cells (1.5 × 10^5^) were seeded in 24-well plates and incubated with or without IC_50_ and IC_80_ concentrations of extracts for 18 hours. Cells were then fixed in 70% ethanol/PBS, pelleted and resuspended in buffer containing 200 *μ*g/mL RNase A and 0.01 mg/mL propidium iodide (PI). The cells were incubated in the dark for 15 minutes at room temperature and then analyzed by FACScan Flow Cytometer (Becton Dickinson, San Jose, Calif, USA). The cell distribution in each phase of the cell cycle was determined using WinMDI software, including subG_1_-peak of apoptotic cells.

### 2.8. Statistical Analysis

To evaluate the statistical significance of the difference of all the values, statistical analysis was performed on the means of the triplicates of at least three independent experiments using a two-tailed Student's *t*-test. *P* values less than .05 were considered significant for all tests.

## 3. Results

### 3.1. Influence of the Genus *Sinularia* Extracts on Cells Adhesion

To investigate six soft corals of the genus *Sinularia* extracts (*S*. *grandilobata*, *S*. *parva*, *S*. *triangula*, *S*. *scabra*, *S*. *nanolobata* and *S*. *gibberosa*) inhibited SCC25 and HaCaT cells adhesion, cells were treated with different concentrations (0, 1, 5, 10, 20, 40, 60, and 100 *μ*g/mL) of extracts for 18 hours, and the cell adhesion assay was performed. Cells remained firmly attached to the culture dish at low concentrations (<40 *μ*g/mL) of extract, and a cytotoxic effect was not observed until almost 70% as indicated in Table 1. The number of attached cells decreased with rising concentrations of extracts (60–100 *μ*g/mL). This reveals that high concentration of extracts may affect cell adhesion on collagen fibers, thus increasing cell cytotoxicity. The cell adhesion assay shows that the extract of *S*. *parva* was the most effective inhibitor of cell survival and adhesion. However, cell adhesion alone does not indicate that a cell is alive. An enzymatic test such as MTS assay is required to further evaluate the effect of extracts on cell cytotoxicity.

### 3.2. Growth-Inhibition Assay Effect of the Genus *Sinularia* Extracts

**Figure 1:**
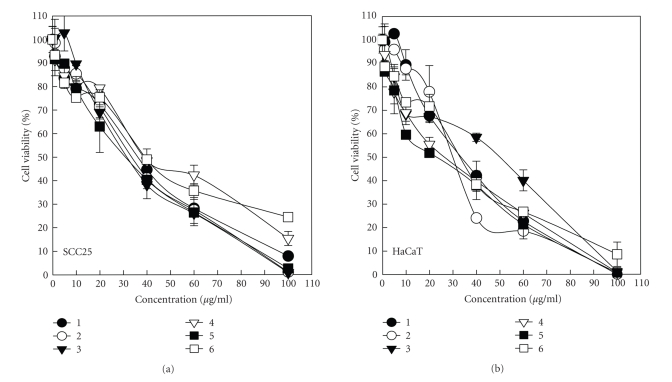
Dose-dependency effects of the genus *Sinularia* extracts on the cell growth inhibition of SCC25 and HaCaT cells. Percentage of viable in cells treated with 0–100 *μ*g/mL concentrations of *Sinularia* extracts for 18 hours and determined by MTS assay. Data are means ± S.D. from three independent experiments. (•) *S*. *grandilobata*, 1; (∘) *S*. *parva*, 2; (▾) *S*. *triangula*, 3; (▿) *S*. *scabra*, 4; (▪) *S*. *nanolobata*, 5; and (□) *S*. *gibberosa*, 6.

MTS assay was conducted to examine the relationship between concentrations of the genus *Sinularia* extracts and the cytotoxicity of SCC25 and HaCaT cells. Cells were treated with extracts at increasing concentrations of 0–100 *μ*g/mL for 18 hours, and the percentage of cell viability was analyzed. Organism extracts were dissolved in DMSO, and a parallel experiment demonstrated that the final concentration of DMSO in the medium (0.1%) did not produce any impact on SCC25 and HaCaT cell cytotoxicity (data not shown). As revealed in Figure 1, all of the extracts inhibited SCC25 and HaCaT cell growth in a dose-dependent manner. The concentrations of extracts causing 50% and 80% cell growth inhibition (IC_50_ and IC_80_) were determined and are presented in Table 2. The (IC_50_)s of *S*. *grandilobata*, *S*. *parva*, *S*. *triangula*, *S*. *scabra*, *S*. *nanolobata* and *S*. *gibberosa* were approximately 36.7l, 34.0, 32.2, 38.9, 31.4, and 39.1 *μ*g/mL for SCC25, and 33.6, 30.3, 49.1, 26.8, 22.6, and 32.9 *μ*g/mL for HaCaT cells. The (IC_80_)s of six extracts were about 75.9, 71.3, 68.7, 93.0, 70.7, and 127.1 *μ*g/mL for SCC25, and 64.7, 54.2, 80.6, 70.5, 62.5, and 74.7 *μ*g/mL for HaCaT cells. The cell cytotoxicity assay demonstrates that *S*. *parva* and *S*. *nanolobata* exhibited the highest potency in inhibiting cell growth, and the results are corresponded to observe with cell adhesion assay.

### 3.3. Impact of the Genus *Sinularia* Extracts on Cell Morphology Changes

**Figure 2:**
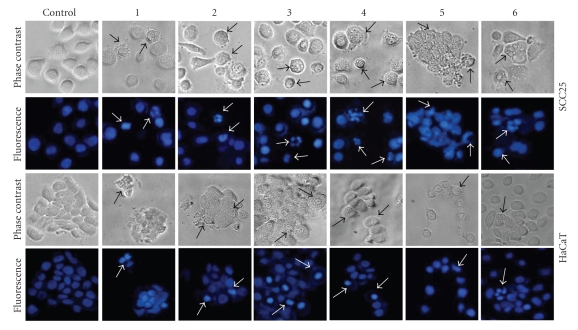
Morphological changes in SCC25 and HaCaT cells after the genus *Sinularia* extracts treatment. A constant concentration of extracts (IC_50_) was added to the cells for 18 hours. The change was inspected by a phase-contrast-inverted microscopy. The cells were then fixed in 4% paraformaldehyde and DNA stained with Hoechst. The nuclei of the cells were visualized using a fluorescent microscope (200×). *S*. *grandilobata*, 1; *S*. *parva*, 2; *S*. *triangula*, 3; *S*. *scabra*, 4; *S*. *nanolobata*, 5; and *S*. *gibberosa*, 6.

A morphological study of SCC25 and HaCaT cells was undertaken to obtain additional information about the cytotoxicity of soft corals of the genus *Sinularia* extracts. Rounding was observed following incubation with extract under concentration of IC_50_ for 18 hours to observe the morphological alterations in the cells. Some sensitive cells were then detached from the surface, and membrane blebbing was shown by using a phase-contrast-inverted microscope. The typical nuclear condensation, nuclear fragmentation, nuclear shrinking, and apoptotic bodies of the cells were then demonstrated by Hoechst staining (see Figure 2). Results of these experiments indicate that the genus *Sinularia* extracts cause apoptosis of human SCC25 and HaCaT cells.

### 3.4. Influence of the Genus *Sinularia* Extracts on Cell-Cycle Distribution and Apoptosis

**Figure 3:**
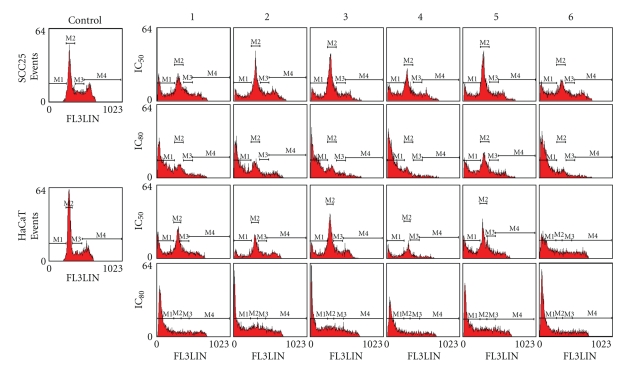
Effect of the genus *Sinularia* extracts on SCC25 and HaCaT cells apoptosis. Flow cytometric analysis of the cell cycle distribution of cells after treatment with extracts (IC_50_ and IC_80_) for 18 hours as described in Materials and Methods. *S*. *grandilobata*, 1; *S*. *parva*, 2; *S*. *triangula*, 3; *S*. *scabra*, 4; *S*. *nanolobata*, 5 and *S*. *gibberosa*, 6.

The cell cycle distribution of SCC25 and HaCaT cells was analyzed with flow cytometry after exposure to the genus *Sinularia* extracts (see Figure 3). Results of treatment of cells with IC_50_ and IC_80_ concentrations of extracts reveal that the main character of apoptosis is the cleavage of nuclear DNA into multiple fragments and reflected G_0_/G_1_ and S-G_2_/M phase together with a dose-dependent increase in sub-G_1_ phase (corresponding to apoptotic cells). As shown in Table 3, the percentage of G_0_/G_1_, S, and G_2_/M phases in SCC25 cells incubated with extracts (IC_80_) for 18 hours was, respectively, 23.7–77.2%, 18.2–64.6%, and 11.4–37.9% less than those in control cells. The sub-G_1_ fraction of apoptotic SCC25 cells following incubation with (IC_80_)-treated cells was around 36.8–79.6 times that of control cells. The percentage of sub-G_1_ phases in (IC_80_)-treated HaCaT cells was approximately 65.3–91.1 times that in untreated control cells. The G_0_/G_1_ and G_2_/M phase fractions were, respectively, about 9.2–25.7% and 46.0–76.4% less in the (IC_80_)-treated cells than in the control cells, and not much change in the S phase populations. The apoptotic cell death induced by treatment with *Sinularia* extracts by flow cytometry was thus very similar to that seen with Hoechst staining (see Figure 2). These results show that the genus *Sinularia* extracts-mediated inhibition of SCC25 and HaCaT cells viability might predominantly induce cells from the G_0_/G_1_ and G_2_/M phases to apoptosis.

## 4. Discussion

Oral cancer, which is one of the most disfiguring cancers, may lead to facial distortion. It is also known to exhibit field cancerization, resulting in development of second primary tumors [12]. Consequently, the development of new antioral cancer drugs, and study of their medicinal value, has become highly significant. The marine environment is a major reservoir of bioactive natural products with potential biomedical application; several marine natural products are seen as potential sources of therapeutic agents for the treatment of multiple disease categories. The majority of marine natural products and their derivatives are formed from invertebrates including soft corals, sponges, tunicates, mollusks, or bryozoans and are currently in advanced preclinical evaluation [1]. However, relatively few attempts have been made to explore resources of structurally unique chemistry for cytotoxic mechanism. This study presents the action mechanism of soft corals of the genus *Sinularia* extracts (*S*. *grandilobata*, *S*. *parva*, *S*. *triangula*, *S*. *scabra*, *S*. *nanolobata* and *S*. *gibberosa*) in the most common type of human oral squamous cell carcinomas SCC25 cells and human premalignant keratinocytes HaCaT cells. The cell adhesion and cell viability assays demonstrate the cytotoxicity effects of six extracts on both cells. Extracts induced morphological changes of chromatin condensation, DNA fragmentation, and sub-G_1_ peak in a DNA histogram of SCC25 and HaCaT cells, indicating cell death by apoptosis.

The previously characterized genus *Sinularia* of secondary metabolites is mainly chemicals that are structurally related to terpenoids. Previous studies have indicated that sinugrandisterols A-D, trihydroxysteroids, and oxygenated terpenoids from the *S*. *grandilobata* impede the proliferation of different cancer cell lines, such as human liver carcinoma (HepG2 and Hepa59T/VGH), human breast cancer cells (MCF-7 and MDA-MB-231), human oral epidermoid carcinoma (KB), and human lung cancer cells (A549) [16]. Three norcembrane-base diterpenoids, leptocladolide A, 1-*epi*-leptocladolide A, 7*E*-leptocladolide A and ineleganoid were isolated from Taiwanese soft coral *S*. *parva*, and these compounds have been revealed to exhibit significant cytotoxic activity against KB and Hepa59T/VGH cancer cell line [17]. Four amphilectane-type diterpenoids, sinulobatins A-D [18], two norsesquiterpenoids, nanonorcaryophyllenes A-B, two diterpenoids, nanolobatins A-B, nordoterpenoids nanolobatin C, (+)-5-hydroxymethyl-5-methylfuran-2-one, and (+)-5-acetoxymethyl-5-methylfuran-2-one were isolated from the *S*. *nanolobata*. Sinulobatins A-D and nanolobatins A-B exhibited moderate cytotoxicity against KB cancer cells. Nanonorcaryophyllenes A-B and nanolobatin C demonstrated no significant cytotoxicity against the tested cell lines, such as KB cells [19]. Additionally, previous reports have shown that polyoxygenated sterols from the formosan soft coral *Sinularia gibberosa* significantly inhibit the upregulation of the proinflammatory inducible nitric oxide synthase (iNOS) and cyclooxygenase-2 (COX-2) proteins of LPS (lipopolysaccharide)-stimulated RAW264.7 macrophage cells and cytotoxic activity against HepG2 (human liver carcinoma), MCF-7, MDA-MB-23 (human breast carcinoma), and A549 (human lung carcinoma) cells [20]. Extracts of soft corals *Sinularia compressa* have been adopted to explore the antibacterial potential of *Bacillus pumilus* and *Pseudomonas vesicularis* [21]. In this work, cell adhesion and cytotoxicity assay indicated that soft corals of the genus *Sinularia* extracts prevented SCC25 and HaCaT cell growth in a concentration-dependent manner. Moreover, the extracts of *S*. *parva* and *S*. *nanolobata* were found to be more effective inhibitors of cell viability than *S*. *grandilobata*, *S*. *triangula*, *S*. *scabra,* and *S*. *gibberosa*, suggesting the *S*. *parva* and *S*. *nanolobata* extracts could be investigated in the further to forage for a potential antioral cancer compounds. In the previous research, it was found that cembranoids extracted from *S*. *parva* and *S*. *nanolobata* showed cytotoxicity in some cell lines [17–19]. Nevertheless, the intrinsic structure and properties of these six soft corals are still not to be clarified. Additionally, the relation yield of compounds purified form soft corals is too few to carry out apoptosis experiments. This study is a preliminary test for cytotoxic activity of soft corals, and very few correlated researches could be found. At least, these results could provide the useful information to determine whether it is worthy to further isolate the natural product or not.

As previously reported, acylspermidines from the soft coral, *Sinularia* Sp. showed potent cytotoxicity against A431 cells [22] and NAKATA cells [23], and induced apoptotic DNA fragmentation and condensation of chromatin in A431 cells obtained from SCC [24]. In this study, morphologic alterations, nuclear chromatin condensation, and formation of apoptotic bodies indicate that extracts of soft corals of the genus *Sinularia* are cytotoxic. The cell cycle distribution demonstrates that extracts sensitized the cells in the G_0_/G_1_ and G_2_/M phases with a concomitant significant increase in the sub-G_1_fraction. Experimental results of this work indicate that extracts from soft corals of the genus *Sinularia* kill not only SCC25, but also HaCaT cells through apoptosis. In summary, these studies demonstrate that the soft corals of the genus *Sinularia* extracts could be a warrant further research investigating the possible antioral cancer compounds in these medicinal marine organisms of soft corals.

## Figures and Tables

**Table 1:** Percentage of SCC25 and HaCaT cells adhesion by different concentrations of the genus *Sinularia* extracts.

Cell lines	Treatment (*μ*g/mL)	No. 1	2	3	4	5	6
SCC25	0	100.0 ± 0.9	100.0 ± 1.0	100.0 ± 0.6	100.0 ± 3.4	100.0 ± 2.2	100.0 ± 1.0
	1	74.7 ± 1.0	118.5 ± 0.9	105.1 ± 8.7	94.1 ± 5.8	110.9 ± 7.7	101.7 ± 6.7
	5	70.4 ± 1.9	71.3 ± 4.8	79.7 ± 2.9	94.0 ± 1.9	70.4 ± 6.7	94.9 ± 2.5
	10	70.4 ± 1.9	70.4 ± 3.8	60.3 ± 3.8	81.4 ± 2.9	70.4 ± 1.9	88.2 ± 8.6
	20	71.3 ± 2.9	69.6 ± 6.7	59.5 ± 6.7	99.1 ± 3.8	71.3 ± 8.6	88.1 ± 8.5
	40	70.4 ± 3.8	66.2 ± 2.9	60.3 ± 3.8	81.4 ± 2.9	71.3 ± 0.9	81.6 ± 2.9
	60	53.5 ± 7.3	38.3 ± 0.1	51.0 ± 2.9	51.0 ± 6.7	66.2 ± 2.9	63.7 ± 1.9
	100	41.7 ± 1.9	21.5 ± 0.3	44.3 ± 0.9	53.5 ± 1.5	55.2 ± 3.8	49.3 ± 8.7

HaCaT	0	100.0 ± 1.8	100.0 ± 1.6	100.0 ± 3.4	100.0 ± 0.9	100.0 ± 6.2	100.0 ± 5.4
	1	78.4 ± 1.8	89.9 ± 2.3	95.7 ± 2.5	99.3 ± 3.7	97.4 ± 4.5	100.0 ± 2.7
	5	80.4 ± 0.9	91.9 ± 3.7	97.5 ± 3.4	95.9 ± 0.9	88.9 ± 7.2	84.3 ± 5.3
	10	79.1 ± 0.9	83.1 ± 7.6	93.8 ± 0.1	88.6 ± 1.8	86.3 ± 9.8	74.5 ± 1.8
	20	80.4 ± 8.2	83.8 ± 8.6	80.9 ± 0.8	79.9 ± 2.8	82.4 ± 1.8	69.3 ± 1.8
	40	77.7 ± 2.9	81.1 ± 1.8	74.7 ± 2.5	75.2 ± 3.7	79.8 ± 1.8	69.3 ± 5.4
	60	75.0 ± 4.6	44.7 ± 7.4	63.6 ± 0.9	75.9 ± 2.0	73.9 ± 1.7	70.6 ± 0.5
	100	64.2 ± 8.8	43.3 ± 9.3	60.5 ± 0.2	61.8 ± 2.9	45.8 ± 4.4	51.7 ± 4.5

(i) Results are the average of three independent experiments. (ii) *S. grandilobata*, 1; *S. parva*, 2; *S. triangula*, 3; *S. scabra*, 4; *S. nanolobata*, 5; *S. gibberosa*, 6.

**Table 2:** Cell viability of the genus *Sinularia* extracts in SCC25 and HaCaT cells.

No.	Cell lines			
	SCC25		HaCaT	
	IC_50_ (*μ*g/mL)	IC_80_ (*μ*g/mL)	IC_50_ (*μ*g/mL)	IC_80_ (*μ*g/mL)
1	36.7 ± 5.6	75.9 ± 3.3	33.6 ± 3.1	64.7 ± 1.8
2	34.0 ± 2.5	71.3 ± 0.9	30.3 ± 4.2	54.2 ± 2.9
3	32.2 ± 2.9	68.7 ± 1.8	49.1 ± 2.3	80.6 ± 3.7
4	38.9 ± 1.5	93.0 ± 4.2	26.8 ± 5.0	70.5 ± 3.6
5	31.4 ± 6.8	70.7 ± 3.1	22.6 ± 2.8	62.5 ± 4.8
6	39.1 ± 1.9	127.1 ± 8.7	32.9 ± 3.7	74.7 ± 1.5

(i) Results are the average of three independent experiments. (ii) *S. grandilobata*, 1; *S. parva*, 2; *S. triangula*, 3; *S. scabra*, 4; *S. nanolobata*, 5; *S. gibberosa*, 6.

**Table 3:** The changes of cell cycle distribution of the genus *Sinularia* extracts in SCC25 and HaCaT cells.

Cell lines	No.	Treatment (*μ*g/mL)	Sub-G_1_ phase		G_0_/G_1_ phase		S phase		G_2_/M phase	
			Mean	%	Mean	%	Mean	%	Mean	%
SCC25	Control	0	1.0	100.0	52.3	100.0	19.8	100.0	27.2	100.0
	1	IC_50_	32.5	3250.0	37.7	72.1	16.5	83.3	13.8	50.7
		IC_80_	64.1	6410.0	21.9	41.9	7.3	36.9	6.8	25.0
	2	IC_50_	18.7	1870.0	46.8	89.4	15.7	79.3	19.8	72.8
		IC^80^	58.9	5890.0	24.1	46.1	8.3	41.9	8.8	32.4
	3	IC_50_	23.6	2360.0	54.3	103.8	12.2	61.6	10.5	38.6
		IC^80^	69.8	6980.0	18.6	35.6	6.8	34.4	4.8	17.6
	4	IC_50_	33.4	3340.0	37.7	72.1	14.4	72.7	14.8	54.4
		IC_80_	80.1	8010.0	13.4	25.6	3.6	18.2	3.1	11.4
	5	IC^50^	24.3	2430.0	51.1	97.7	12.5	63.1	12.5	46.0
		IC_80_	36.8	3680.0	40.4	77.2	12.8	64.6	10.3	37.9
	6	IC^50^	33.7	3370.0	32.8	62.7	15.5	78.3	18.3	67.3
		IC_80_	79.6	7960.0	12.4	23.7	4.4	22.2	3.6	13.2

HaCaT	Control	0	0.8	100.0	59.5	100.0	16.2	100	23.7	100.0
	1	IC^50^	35.3	4412.5	34.3	57.6	13.1	80.9	16.7	70.5
		IC_80_	69.4	8675.0	6.7	11.3	7.9	48.8	16.1	67.9
	2	IC_50_	19.4	2425.0	42.8	71.9	17.8	109.9	20.3	85.7
		IC_80_	52.3	6537.5	15.3	25.7	15.1	93.2	17.4	73.4
	3	IC_50_	25.1	3137.5	45.2	76.0	16.4	101.2	13.9	58.6
		IC^80^	56.1	7012.5	13.2	22.2	16.1	100	15.1	63.7
	4	IC^50^	39.1	4887.5	38.2	64.2	13.1	80.9	9.6	40.5
		IC_80_	71.3	8912.5	8.1	13.6	9.9	61.1	10.9	46.0
	5	IC_50_	35.8	4475.0	35.4	59.5	15.4	95.1	13.8	58.2
		IC_80_	58.9	7362.5	11.9	20.0	14.3	88.3	15.1	63.7
	6	IC^50^	57.4	7175.0	10.2	17.1	12.8	79.0	19.7	83.1
		IC_80_	72.9	9112.5	5.5	9.2	8.3	51.2	13.3	56.1

(i) *S. grandilobata*, 1; *S. parva*, 2; *S. triangula*, 3; *S. scabra*, 4; *S. nanolobata*, 5; *S. gibberosa*, 6.
